# Supplementary material for: Regulatory chromatin rewiring promotes metabolic switching during adaptation to oncogenic receptor tyrosine kinase inhibition
Source: Oncogene. Author manuscript; Available in PMC 2022 Oct 24. (PMC9586873; doi:10.1038/s41388-022-02465-w)
Supplement: Supplementary file 1 [file EMS153561-supplement-Supplementary_file_1.docx]

**Oesophageal Cancer Clinical and Molecular Stratification (OCCAMS) Consortium**:

Rebecca C. Fitzgerald^1^, Paul A.W. Edwards^1,2^, Nicola Grehan^1^, Barbara Nutzinger^1^, Elwira Fidziukiewicz^1^, Aisling M Redmond^1^, Sujath Abbas^1^, Adam Freeman^1^ Elizabeth C. Smyth^5^, Maria O’Donovan^1,3^, Ahmad Miremadi^1,3^, Shalini Malhotra^1,3^, Monika Tripathi^1,3^, Calvin Cheah^1^, Hannah Coles^1^, Conor Flint^1^, Matthew Eldridge^2^, Maria Secrier^2^, Ginny Devonshire^2^, Sriganesh Jammula^2^, Jim Davies^4^, Charles Crichton^4^, Nick Carroll^5^, Richard H.Hardwick^5^, Peter Safranek^5^, Andrew Hindmarsh^5^, Vijayendran Sujendran^5^, Stephen J. Hayes^6,13^, Yeng Ang^6,7,26^, Andrew Sharrocks^26^, Shaun R. Preston^8^, Izhar Bagwan^8^, Vicki Save^9^, Richard J.E. Skipworth^9^, Ted R. Hupp^20^, J. Robert O’Neill^5,9,20^, Olga Tucker^10,29^, Andrew Beggs^10,25^, Philippe Taniere^10^, Sonia Puig^10^, Gianmarco Contino^10^, Timothy J. Underwood^11,12^, Robert C. Walker^11,12^, Ben L. Grace^11^, Jesper Lagergren^14,22^, James Gossage^14,21^, Andrew Davies^14,21^, Fuju Chang^14,21^, Ula Mahadeva^14^, Vicky Goh^21^, Francesca D. Ciccarelli^21^, Grant Sanders^15^, Richard Berrisford^15^, David Chan^15^, Ed Cheong^16^, Bhaskar Kumar^16^, L. Sreedharan^16^ Simon L Parsons^17^, Irshad Soomro^17^, Philip Kaye^17^, John Saunders^6, 17^, Laurence Lovat^18^, Rehan Haidry^18^, Michael Scott^19^, Sharmila Sothi^23^, Suzy Lishman^2^, George B. Hanna^27^, Christopher J. Peters^27^,Krishna Moorthy^27^, Anna Grabowska^28^, Richard Turkington^30^, Damian McManus^30^, Helen Coleman^30^, Russell D Petty^31^ , Freddie Bartlett^32^

^1^ Medical Research Council Cancer Unit, Hutchison/Medical Research Council Research Centre, University of Cambridge, Cambridge, UK

^2^ Cancer Research UK Cambridge Institute, University of Cambridge, Cambridge, UK

^3^ Department of Histopathology, Addenbrooke’s Hospital, Cambridge, UK

^4^Department of Computer Science, University of Oxford, UK, OX1 3QD

^5^Cambridge University Hospitals NHS Foundation Trust, Cambridge, UK, CB2 0QQ

^6^Salford Royal NHS Foundation Trust, Salford, UK, M6 8HD

^7^Wigan and Leigh NHS Foundation Trust, Wigan, Manchester, UK, WN1 2NN

^8^Royal Surrey County Hospital NHS Foundation Trust, Guildford, UK, GU2 7XX

^9^Edinburgh Royal Infirmary, Edinburgh, UK, EH16 4SA

^10^University Hospitals Birmingham NHS Foundation Trust, Birmingham, UK, B15 2GW

^11^University Hospital Southampton NHS Foundation Trust, Southampton, UK, SO16 6YD

^12^Cancer Sciences Division, University of Southampton, Southampton, UK, SO17 1BJ

^13^Faculty of Medical and Human Sciences, University of Manchester, UK, M13 9PL

^14^ Guy’s and St Thomas’s NHS Foundation Trust, London, UK, SE1 7EH

^15^Plymouth Hospitals NHS Trust, Plymouth, UK, PL6 8DH

^16^Norfolk and Norwich University Hospital NHS Foundation Trust, Norwich, UK, NR4 7UY

^17^Nottingham University Hospitals NHS Trust, Nottingham, UK, NG7 2UH

^18^University College London, London, UK, WC1E 6BT

^19^Wythenshawe Hospital, Manchester, UK, M23 9LT

^20^Edinburgh University, Edinburgh, UK, EH8 9YL

^21^King’s College London, London, UK, WC2R 2LS

^22^Karolinska Institute, Stockholm, Sweden, SE-171 77

^23^University Hospitals Coventry and Warwickshire NHS, Trust, Coventry, UK, CV2 2DX

^24^Peterborough Hospitals NHS Trust, Peterborough City Hospital, Peterborough, UK, PE3 9GZ

^25^Institute of Cancer and Genomic sciences, University of Birmingham, B15 2TT

^26^Faculty of Biology, Medicine and Health, University of Manchester, UK, M13 9PL.

^27^Department of Surgery and Cancer, Imperial College, London, UK, W2 1NY

^28^Queen’s Medical Centre, University of Nottingham, Nottingham, UK

^29^Heart of England NHS Foundation Trust, Birmingham, UK, B9 5SS.

^30^Centre for Cancer Research and Cell Biology, Queen’s University Belfast, Northern Ireland BT7 1NN.

^31^Tayside Cancer Centre, Ninewells Hospital and Medical School, Dundee, DD1 9SY

32 Portsmouth Hospitals NHS Trust, Portsmouth, PO6 3LY
